# Supplementary material for: Interferon-epsilon is a novel regulator of NK cell responses in the uterus
Source: EMBO Mol Med. 2024 Jan 23;16(2):267–93. doi: 10.1038/s44321-023-00018-6 (PMC10897320; doi:10.1038/s44321-023-00018-6)
Supplement: Supplementary file 1 — Table EV1 [file 44321_2023_18_MOESM1_ESM.docx]

**Table EV1.** **Antibody details**

| **Antigen** | **Format** | **Cat #** | **Manufacturer** | **Clone** | **Dilution** |
| --- | --- | --- | --- | --- | --- |
| CD16/32 | Fc block | CUS-HB-197 | BioXCell | 2.4G2 | 10ng/mL |
| CD45 | PerCP | 103130 | Biolegend | 30-F11 | 1:100 |
| CD3 | APC | 100311 | Biolegend | 145-2C11 | 1:400 |
| NK1.1 | PE-Cy7 | 108714 | Biolegend | PK136 | 1:100 |
| CD49b | FITC | 108905 | Biolegend | DX5 | 1:20 |
| CD122 | Biotin | 105904 | Biolegend | 5H4 | 1:50 |
| CD69 | FITC | 104506 | Biolegend | H1.2F3 | 1:50 |
| IFNγ | PE | 505808 | Biolegend | XMG1.2 | 1:100 |
| N/A | Isotype control | 400407 | Biolegend | RTK2071 | 1:100 |
| CD3 | AF700 | 100216 | Biolegend | 17A2 | 1:100 |
| B220 | PE | 103208 | Biolegend | RA3-6B2 | 1:100 |
| CD11b | PerCP | 101229 | Biolegend | M1/70 | 1:100 |
| CD69 | PECF594 | 562455 | BD | H1.2F3 | 1:100 |
| CD45 | FITC | 103108 | Biolegend | 30-F11 | 1:200 |
| CD3 | APC-Cy7 | 100330 | Biolegend | 145-2C11 | 1:200 |
| CD4 | APC-Cy7 | 100525 | Biolegend | RM4-5 | 1:200 |
| GR1 | APC-Cy7 | 108423 | Biolegend | RB6-8C5 | 1:50 |
| B220 | APC-Cy7 | 561102 | BD | RA3-6B2 | 1:50 |
| C-kit | BV711 | 105835 | Biolegend | 2B8 | 1:50 |
| Sca-1 | BV510 | 108129 | Biolegend | D7 | 1:100 |
| FLT3 | PE | 135305 | Biolegend | A2F10 | 1:50 |
| IL-7Rα | BV421 | 566300 | BD | SB/199 | 1:50 |
| CD122 | BV650 | 740509 | BD | 5H4 | 1:50 |
| NKG2D | Biotin | 115703 | Biolegend | C7 | 1:20 |
| CD49b | APC | 108909 | Biolegend | DX5 | 1:100 |
| CD11b | AF700 | 557960 | BD | M1/70 | 1:100 |
| IFNγ | BV711 | 564336 | BD | XMG1.2 | 1:100 |
| N/A | Isotype control | 563283 | BD | R3-34 | 1:100 |
| CD69 | BUV395 | 740220 | BD | H1.2F3 | 1:50 |
| CD45 | BV510 | 563891 | BD | 30-F11 | 1:100 |
| CD11b | APC-Cy7 | 101225 | Biolegend | M1/70 | 1:500 |
| CD11c | PE-Cy7 | 25-0114-82 | eBiosciences | N418 | 1:500 |
| NKp46 | BV711 | 137621 | Biolegend | 29A1.4 | 1:50 |
| Ly6G | Pacific Blue | 127612 | Biolegend | 1A8 | 1:50 |
| MHC-II | AF700 | 56-5321-82 | eBioscience | M5/114.15.2 | 1:200 |
| CD19 | BV650 | 115541 | Biolegend | 6D5 | 1:100 |
| CD4 | BV785 | 100551 | Biolegend | RM4-5 | 1:200 |
| CD8 | APC-Cy7 | 100714 | Biolegend | 53-6.7 | 1:100 |
| Pan Cytokeratin | PE | AB52460 | Abcam | C-11 | 1:50 |
| GFP | AF488 | A-21311 | Invitrogen | Rabbit polyclonal | 1:2000 |
| IFNε | APC | HE70 | In house (SS. Lim, in prep.) | Mouse monoclonal IgG2a | 3μg/mL |
| IFNε | Naked | HE70 | In house (SS. Lim, in prep.) | Mouse monoclonal IgG2a | 3μg/mL |
| Mouse IgG | Biotin | BA-9200-1.5 | Vector Laboratories | Goat polyclonal | 7.5µg/mL |
| IL-15 | Neutralizing | ab7213 | Abcam | Rabbit polyclonal | 10µg/mL |
| N/A | Isotype control | ab171870 | Abcam | Rabbit polyclonal | 10µg/mL |
