# Supplementary material for: Interferon-epsilon is a novel regulator of NK cell responses in the uterus
Source: EMBO Mol Med. 2024 Jan 23;16(2):267–93. doi: 10.1038/s44321-023-00018-6 (PMC10897320; doi:10.1038/s44321-023-00018-6)
Supplement: Supplementary file 2 — Table EV2 [file 44321_2023_18_MOESM2_ESM.docx]

**Table EV2.** **Characterization of immune cell populations**

| **Cell type** | **Surface marker expression** | **FSC/SSC profile** |
| --- | --- | --- |
| Leukocyte | CD45^+^ |  |
| Natural killer (NK) cell | CD45^+^CD3^-^NK1.1^+^ | FSC^low-int^ SSC^low^ |
| Uterine (u)NK cell | CD45^+^CD3^-^NK1.1^-^CD49b^-^CD122^+^ | FSC^low-int^ SSC^low^ |
| Pre-pro NK cell | CD45^+^Lin (CD3, B220, GR1, CD11b)^-^FLT3^-^IL-7Rα^+^C-kit^low/-^CD122^-^NK1.1^-^CD49b^-^NKG2D^+^Sca-1^+^ | FSC^low-int^ SSC^low^ |
| Precursor NK cell | CD45^+^Lin^-^FLT3^-^IL-7Rα^+^C-kit^low/-^CD122^+^NK1.1^-^CD49b^-^NKG2D^+^ | FSC^low-int^ SSC^low^ |
| Bone marrow (BM) immature NK cell | CD45^+^Lin^-^CD122^+^NK1.1^+^CD11b^-^ | FSC^low-int^ SSC^low^ |
| BM mature NK cell | CD45^+^CD3^-^CD122^+^NK1.1^+^CD11b^+^ | FSC^low-int^ SSC^low^ |
