# Supplementary material for: Interferon-epsilon is a novel regulator of NK cell responses in the uterus
Source: EMBO Mol Med. 2024 Jan 23;16(2):267–93. doi: 10.1038/s44321-023-00018-6 (PMC10897320; doi:10.1038/s44321-023-00018-6)
Supplement: Supplementary file 3 — Table EV3 [file 44321_2023_18_MOESM3_ESM.docx]

**Table EV3.** **Custom primers used for quantitative PCR**

| **Target** | **Species** | **Strand** | **Nucleotide Sequence** |
| --- | --- | --- | --- |
| *Hprt* | Mouse | Forward | 5’-AGGCCAGACTTTGTTGGATTTGAA-3’ |
|  |  | Reverse | 5’-CAACTTGCGCTCATCTTAGGCTTT-3’ |
| *Il15* | Mouse | Forward | 5’-ACAGCTCAGAGAGGTCAG-3’ |
|  |  | Reverse | 5’-ACCAGCAAGGACCATGAAGAG-3’ |
| *Cxcl10* | Mouse | Forward | 5’-CCAAGTGCTGCCGTCATTTTC-3’ |
|  |  | Reverse | 5’-TCCCTATGGCCCTCATTCTCA-3’ |
| *Il12b* | Mouse | Forward | 5’-CACGTCCTCATGGCTGGTGC-3’ |
|  |  | Reverse | 5’-TGCCCGAGAGTCAGGGGAACT-3’ |
| *Il18* | Mouse | Forward | 5’-TCAGACAACTTTGGCCGACT-3’ |
|  |  | Reverse | 5’-CAGTCTGGTCTGGGGTTCAC-3’ |
| *GAPDH* | Human | Forward | 5’-GTCAAGGCTGAGAACGGGAA-3’ |
|  |  | Reverse | 5’-TCGCCCCACTTGATTTTGGA-3’ |
| *IL15* | Human | Forward | 5’-AACAGAAGCCAACTGGGTGAA-3’ |
|  |  | Reverse | 5’-TGGGGTGAACATCACTTTCCG-3’ |
| *NCR1* | Human | Forward | 5’-CCACAGAGGGACATACCGATG-3’ |
|  |  | Reverse | 5’-TCTCAATGTCGCCTGTGACC-3’ |
| *ompA* | *C. muridarum* | Forward | 5’-GCCGTTTTGGGTTCTGCTT-3’ |
|  |  | Reverse | 5’-CGTCAATCATAAGGCTTGGTTCA-3’ |

*Hprt*: Hypoxanthine phosphoribosyltransferase 1; *Il-*: interleukin-; *Cxcl10*: C-X-C motif chemokine ligand 10; *GAPDH*: Glyceraldehyde 3-phosphate dehydrogenase; *NCR1*: Natural Cytotoxicity Triggering Receptor 1; *ompA*: outer membrane protein A.
