# Supplementary figures and images for: Interferon-epsilon is a novel regulator of NK cell responses in the uterus
Source: EMBO Mol Med. 2024 Jan 23;16(2):267–93. doi: 10.1038/s44321-023-00018-6 (PMC10897320; doi:10.1038/s44321-023-00018-6)

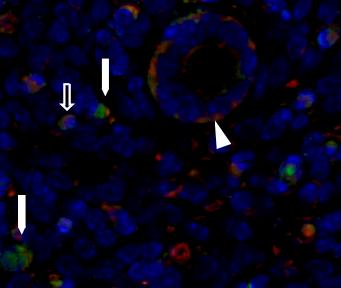

Supplement: Supplementary file 6 — Source Data Fig. 3 [file 44321_2023_18_MOESM6_ESM.zip › Fig 3/Fig 3F - Micrograph panels/Figure 3 F - 20x merged boxed area.tif]

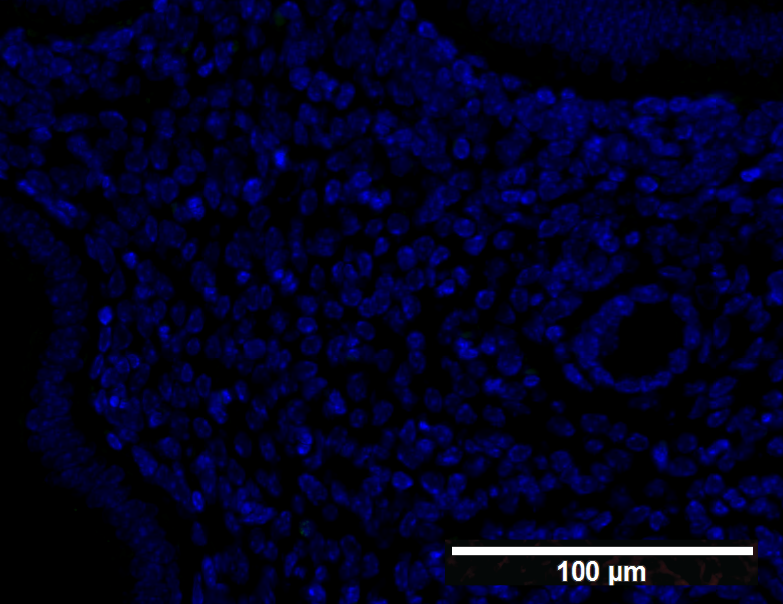

Supplement: Supplementary file 6 — Source Data Fig. 3 [file 44321_2023_18_MOESM6_ESM.zip › Fig 3/Fig 3F - Micrograph panels/Figure 3 F - 20x merged isotype control.tif]

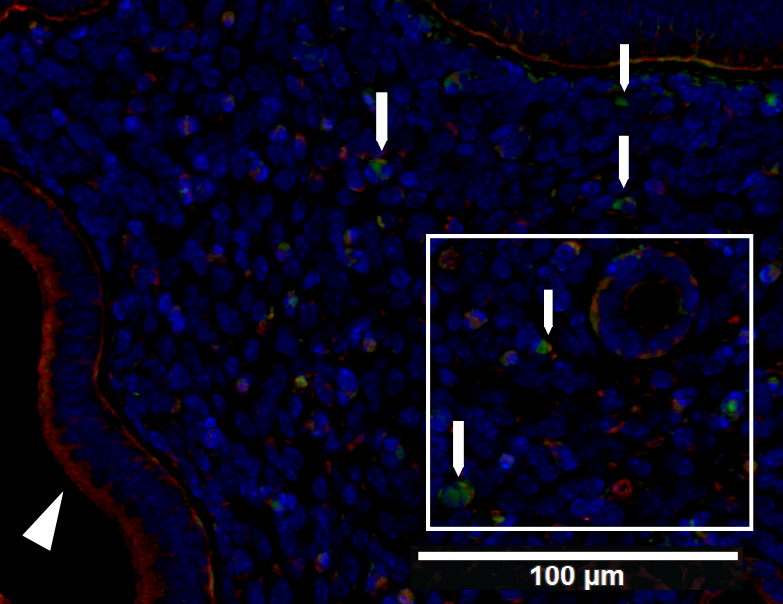

Supplement: Supplementary file 6 — Source Data Fig. 3 [file 44321_2023_18_MOESM6_ESM.zip › Fig 3/Fig 3F - Micrograph panels/Figure 3 F - 20x merged IFNe(red) IL-15(green).tif]
