# Supplementary material for: Interferon-epsilon is a novel regulator of NK cell responses in the uterus
Source: EMBO Mol Med. 2024 Jan 23;16(2):267–93. doi: 10.1038/s44321-023-00018-6 (PMC10897320; doi:10.1038/s44321-023-00018-6)
Supplement: Supplementary file 12 — Expanded View Figures [file 44321_2023_18_MOESM12_ESM.pdf]

Expanded View Figures

Figure EV1. Effect of interferon (IFN)ε deficiency on the frequency of leukocyte populations in the uterus at 3 days and oviduct pathology at 14 days post *Chlamydia* infection and flow cytometry gating strategy for identification of natural killer (NK) cell and progenitor populations.

(**A**) Frequency of natural killer (NK) cells ($FSC^{low-int}$ SSC^{low} $CD3^-$ $NK1.1^+$), macrophages (FSC^{int} SSC^{int} $F480^+$), neutrophils ($FSC^{low-int}$ $SSC^{int-high}$ $F480^-$ $CD11b^+$ $GR1^+$), plasmacytoid dendritic cells (pDCs; $FSC^{low-int}$ $SSC^{low-int}$ $CD11c^+$ $CD11b^-$ $GR1^+$ $PDCA^+$), myeloid (m)DCs ($FSC^{low-int}$ $SSC^{low-int}$ $CD11c^+$ $CD11b^+$ $GR1^-$ $PDCA^-$), $CD4^+$ T cells ($FSC^{low-int}$ SSC^{low} $CD3^+$ $CD4^+$), $CD8^+$ T cells ($FSC^{low-int}$ SSC^{low} $CD3^+$ $CD8^+$), B cells ($FSC^{low-int}$ SSC^{low} $CD3^-$ $B220^+$), and NK T cells ($FSC^{low-int}$ SSC^{low} $CD3^+$ $NK1.1^+$) in uterine horns from *Ifne*$^{-/-}$ and wild-type (WT) C57BL/6 mice on day 3 of *Chlamydia muridarum* infection measured by flow cytometry. (**B**) Flow cytometry of uterine horn cells from *Ifne*$^{-/-}$ and WT mice on day 3 of *C. muridarum* infection showing gating for innate lymphoid cell (ILC) populations (ILC1-3: $FSC^{low-int}$ SSC^{low} $CD45^+$ Lin^- $CD90.2^+$ IL-7Rα$^+$ T-bet$^{+/-}$). (**C**) Cross-sectional area of the oviducts (in mm^2) from *Ifne*$^{-/-}$ and WT mice on day 14 of *C. muridarum* (Cmu) or sham (SPG) infection. (**D–H**) Flow cytometry gating strategy for NK cell populations. (**D**) For all stains doublets and debris were first excluded, leukocytes ($CD45^+$ cells) selected and then lymphocytes selected based on size (forward scatter [FSC]) and granularity (side scatter [SSC]). (**E**) Conventional NK cells ($FSC^{low-int}$ SSC^{low} $CD45^+$ $CD3^-$ $NK1.1^+$) and T cells ($FSC^{low-int}$ SSC^{low} $CD45^+$ $CD3^+$ $NK1.1^-$) were gated based on NK1.1 and CD3 expression. (**F**) $CD3^-$ $NK1.1^-$ cells were gated and tissue-resident uterine (u)NK cells ($FSC^{low-int}$ SSC^{low} $CD45^+$ $CD3^-$ $NK1.1^-$ $CD49b^-$ $CD122^+$) identified based on CD49b and CD122 expression. (**G**) Lineage marker (lin; CD3, CD4, B220, GR1, and CD11b)$^-$ cells were gated followed by $FLT3^-$ and IL-7Rα$^+$ C-kit$^{low/-}$ cells. $CD122^-$ $NK1.1^-$ cells were gated and pre-pro NK cell progenitors ($FSC^{low-int}$ SSC^{low} $CD45^+$ lin^- $FLT3^-$ IL-7Rα$^+$ C-kit$^{low/-}$ $CD122^-$ $NK1.1^-$ $CD49b^-$ $NKG2D^+$ Sca-1$^+$) gated based on CD49b, NKG2D and Sca-1 expression. $CD122^+$ $NK1.1^-$ cells were gated and precursor NK cell progenitors ($FSC^{low-int}$ SSC^{low} $CD45^+$ lin^- $FLT3^-$ IL-7Rα$^+$ C-kit$^{low/-}$ $CD122^+$ $NK1.1^-$ $CD49b^-$ $NKG2D^+$) gated based on CD49b and NKG2D expression. (**H**) In the bone marrow lin^- $CD11b^-$cells were gated and immature NK cells ($FSC^{low-int}$ SSC^{low} $CD45^+$ lin^- $CD11b^-$ $CD122^+$ $NK1.1^+$) gated based on NK1.1 and CD122 expression. Lin^- $CD11b^+$ cells were gated and mature NK cells ($FSC^{low-int}$ SSC^{low} $CD45^+$ lin^- $CD11b^+$ $CD122^+$ $NK1.1^+$) gated based on NK1.1 and CD122 expression. Data information: Data in (**A**) and (**C**) is presented as mean ± SEM, with individual values. $^{**}p < 0.01$, $^{****}p < 0.0001$ ((**A**): two-way ANOVA; (**C**): one-way ANOVA). (**A**): $n = 4$ (data from one experiment), (**C**): $n = 18$-19 (data from two experiments; all biological replicates). For flow cytometry analysis of NK cells, single cell suspensions from (**D–F**) uterine horn, (**D–F**) spleen, (**D**, **G**) lymph node and (**D**, **G**, **H**) bone marrow tissues were stained with various antibodies (**D**, **E**) with or (**D**, **F–H**) without stimulation. (**F**) is repeated in Fig. 1B and pre-pro and precursor NK cell panels in (**G**) are repeated in Fig. 2A, E.

 © The Author(s)

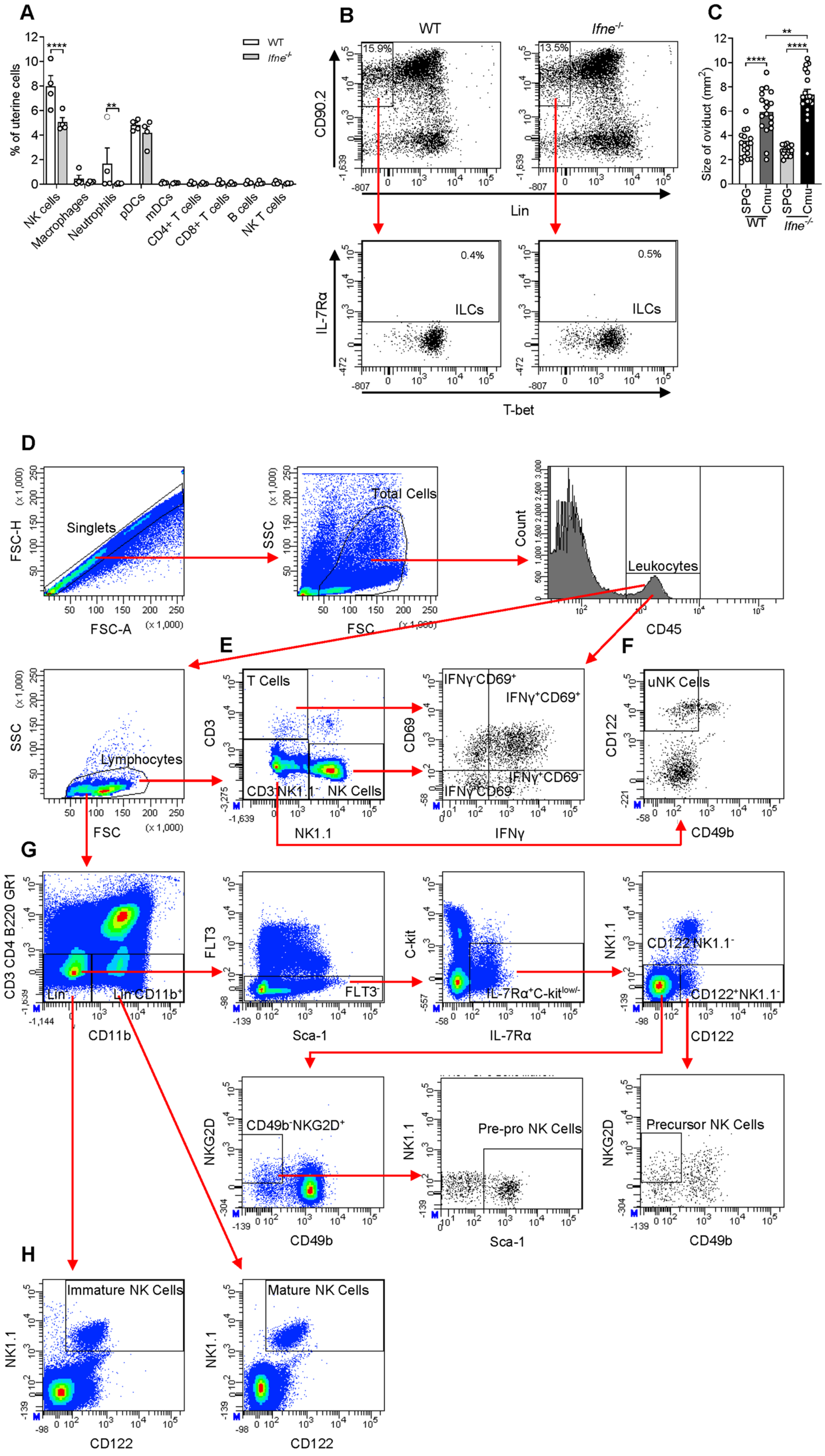
A
% of uterine cells
WT
Ifne-/-
****
**
NK cells
Macrophages
Neutrophils
pDCs
mDCs
CD4+ T cells
CD8+ T cells
B cells
NK T cells
B
WT
Ifne-/-
15.9%
13.5%
CD90.2
Lin
0.4%
0.5%
ILCs
IL-7Rα
T-bet
C
Size of oviduct (mm2)
****
**
SPG
Cmu
WT
Ifne-/-
D
Singlets
FSC-H
FSC-A
Total Cells
SSC
FSC
Count
Leukocytes
CD45
E
Lymphocytes
T Cells
CD3-NK1.1-
NK Cells
CD3
NK1.1
IFNγ-CD69+
IFNγ+CD69+
IFNγ+CD69-
IFNγ-CD69-
CD69
IFNγ
F
uNK Cells
CD122
CD49b
G
CD3 CD4 B220 GR1
Lin-
Lin-CD11b+
CD11b
FLT3
FLT3-
Sca-1
C-kit
IL-7Rα+C-kitlow/-
IL-7Rα
CD122-NK1.1-
CD122+NK1.1-
NK1.1
CD122
CD49b-NKG2D+
NKG2D
CD49b
Pre-pro NK Cells
Sca-1
Precursor NK Cells
NKG2D
H
Immature NK Cells
Mature NK Cells
NK1.1
CD122

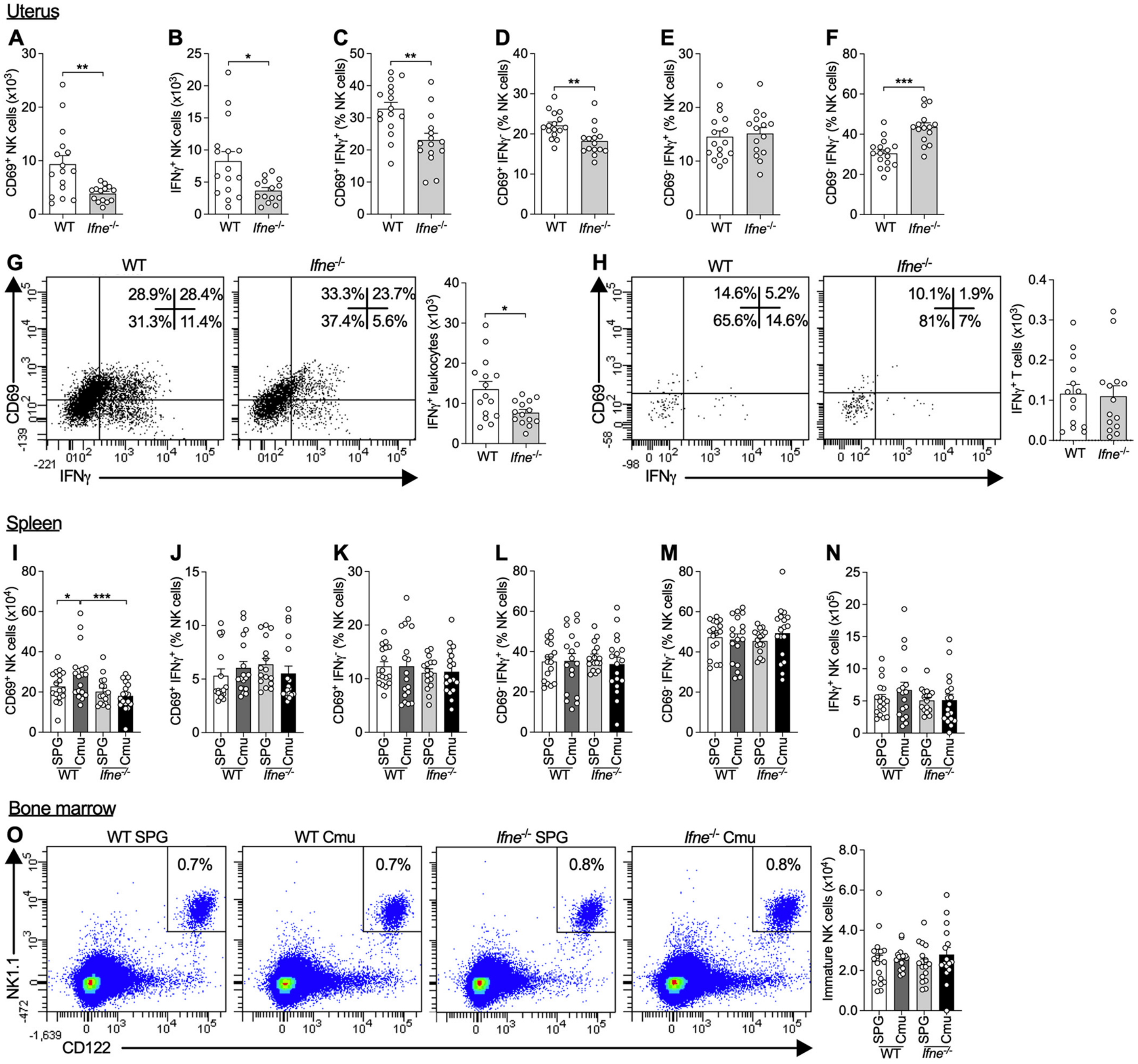

Figure EV2. Effect of interferon (IFN)ε deficiency on the numbers and proportions of activated and IFNγ-producing natural killer (NK) cells in the uterus and spleen, numbers of IFNγ-producing T cells in the uterus, and numbers of immature NK cells in bone marrow.

(**A, B**) Quantification of (**A**) CD69^{+} and (**B**) IFNγ^{+} conventional NK cells (FSC$^{low\text{-}int}$ SSC^{low} CD45^{+} CD3^{-} NK1.1^{+}) in uterine horns from *Ifne*$^{-/-}$ and wild-type (WT) C57BL/6 mice on day 3 of *Chlamydia muridarum* infection measured by flow cytometry. (**C–F**) Frequency of conventional NK cells expressing (**C**) CD69^{+} IFNγ^{+}, (**D**) CD69^{+} IFNγ^{-}, (**E**) CD69^{-} IFNγ^{+}, and (**F**) CD69^{-} IFNγ^{-} in uterine horns. (**G**) Flow cytometry of uterine horn cells showing CD69$^{+/-}$ IFNγ$^{+/-}$ leukocytes (CD45^{+}) and quantification. (**H**) Flow cytometry of uterine horn cells showing CD69$^{+/-}$ IFNγ$^{+/-}$ T cells (FSC$^{low\text{-}int}$ SSC^{low} CD45^{+} CD3^{+}) and quantification. (**I**) Quantification of CD69^{+} conventional NK cells (FSC$^{low\text{-}int}$ SSC^{low} CD45^{+} CD3^{-} NK1.1^{+}) in spleens. (**J–M**) Frequency of conventional NK cells expressing (**J**) CD69^{+} IFNγ^{+}, (**K**) CD69^{+} IFNγ^{-}, (**L**) CD69^{-} IFNγ^{+}, and (**M**) CD69^{-} IFNγ^{-} in spleens. (**N**) Quantification of IFNγ^{+} conventional NK cells (FSC$^{low\text{-}int}$ SSC^{low} CD45^{+} CD3^{-} NK1.1^{+}) in spleens. (**O**) Flow cytometry of bone marrow from femurs showing immature conventional NK cells (FSC$^{low\text{-}int}$ SSC^{low} CD45^{+} lin^{-} CD11b^{-} CD122^{+} NK1.1^{+}) and quantification. Data information: The % displayed on the flow cytometry plots are the % of the parent population the cells within the gates/quadrants comprise. All data presented as mean ± SEM, with individual values. *$p < 0.05$, **$p < 0.01$, ***$p < 0.001$ ((**A–H**): two-tailed Mann–Whitney test; (**I–O**): one-way ANOVA). (**A–H**): $n \geq 15$ (data from three experiments), (**I–O**): $n \geq 15$ (data from two experiments; all biological replicates).

 © The Author(s)

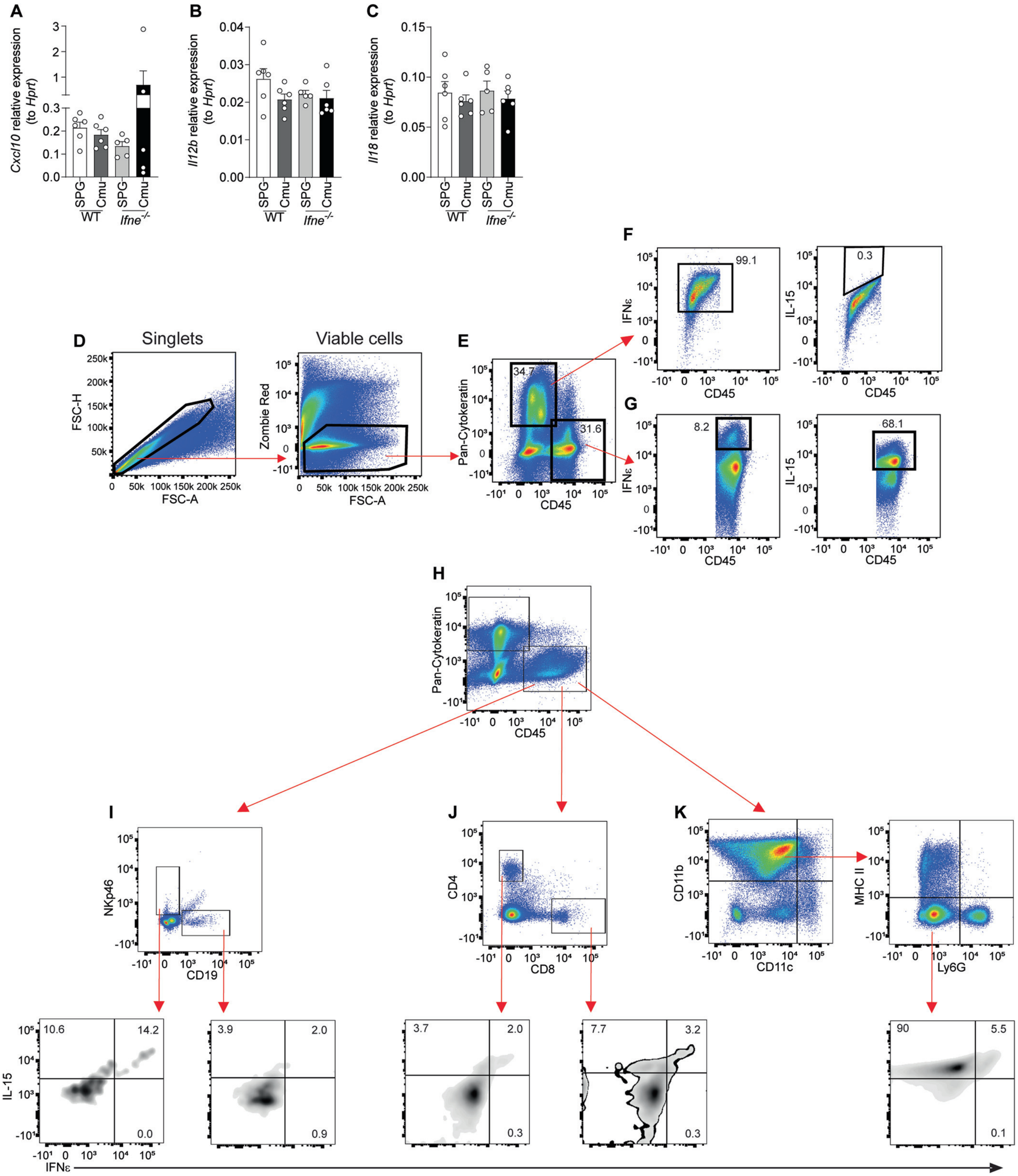
A
B
C
Cxcl10 relative expression (to Hprt)
Il12b relative expression (to Hprt)
Il18 relative expression (to Hprt)
SPG
Cmu
WT
Ifne-/-
D
Singlets
Viable cells
FSC-H
FSC-A
Zombie Red
E
Pan-Cytokeratin
CD45
34.7
31.6
F
99.1
0.3
IFNε
IL-15
G
8.2
68.1
H
I
NKp46
CD19
J
CD4
CD8
K
CD11b
CD11c
MHC II
Ly6G
10.6
14.2
0.0
3.9
2.0
0.9
3.7
2.0
0.3
7.7
3.2
0.3
90
5.5
0.1

Figure EV3. Effect of interferon (IFN)ε deficiency on CXCL10, IL-12, and IL-18 expression in the uterus and flow cytometry gating strategy for identification of interferon (IFN)ε and IL-15 expressing cells in uteri and frequency of co-expression in common immune cell populations.

(**A–C**) qPCR analysis of *Cxcl10* (**A**), *Il12b* (**B**) and *Il18* (**C**) expression normalized to the expression of the housekeeping gene *Hprt* in uterine horns from *Ifne*$^{-/-}$ and wild-type (WT) C57BL/6 mice on day 3 of *Chlamydia muridarum* (Cmu) or sham (SPG) infection. All data presented as mean ± SEM, with individual values (A-C: not significant, one-way ANOVA). (**A–C**): $n \geq 5$ (data from one experiment). (**D**) Single-cell suspensions of uteri from IL-15-CFP reporter mice were stained with antibodies against cell surface markers followed by intracellular staining. Single, viable cells were selected. (**E**) Epithelial cells (Pan-Cytokeratin^{+}) and immune cells (CD45^{+}) were gated based on Pan-Cytokeratin and CD45 expression. (**F**) IFNε and IL-15 expression was gated in epithelial cells and (**G**) immune cells, followed by identification of immune cell type shown in Fig. 3. (**F**, **G**) are repeated in Fig. 3**G**, **H**. (**H–K**) (**H**) To determine frequency of IFNε and IL-15 co-expression in immune cell subsets, CD45^{+} cells were gated for (**I**) NK cells (NKp46^{+}), B cells (CD19^{+}), (**J**) T cells (CD4^{+} or CD8^{+}), and (**K**) monocytes/macrophages (CD11b^{+} CD11c$^{neg\text{-}low}$ MHC-II^{-} Ly6G^{-}) then IFNε and IL-15 expression was characterized these populations.

 © The Author(s)

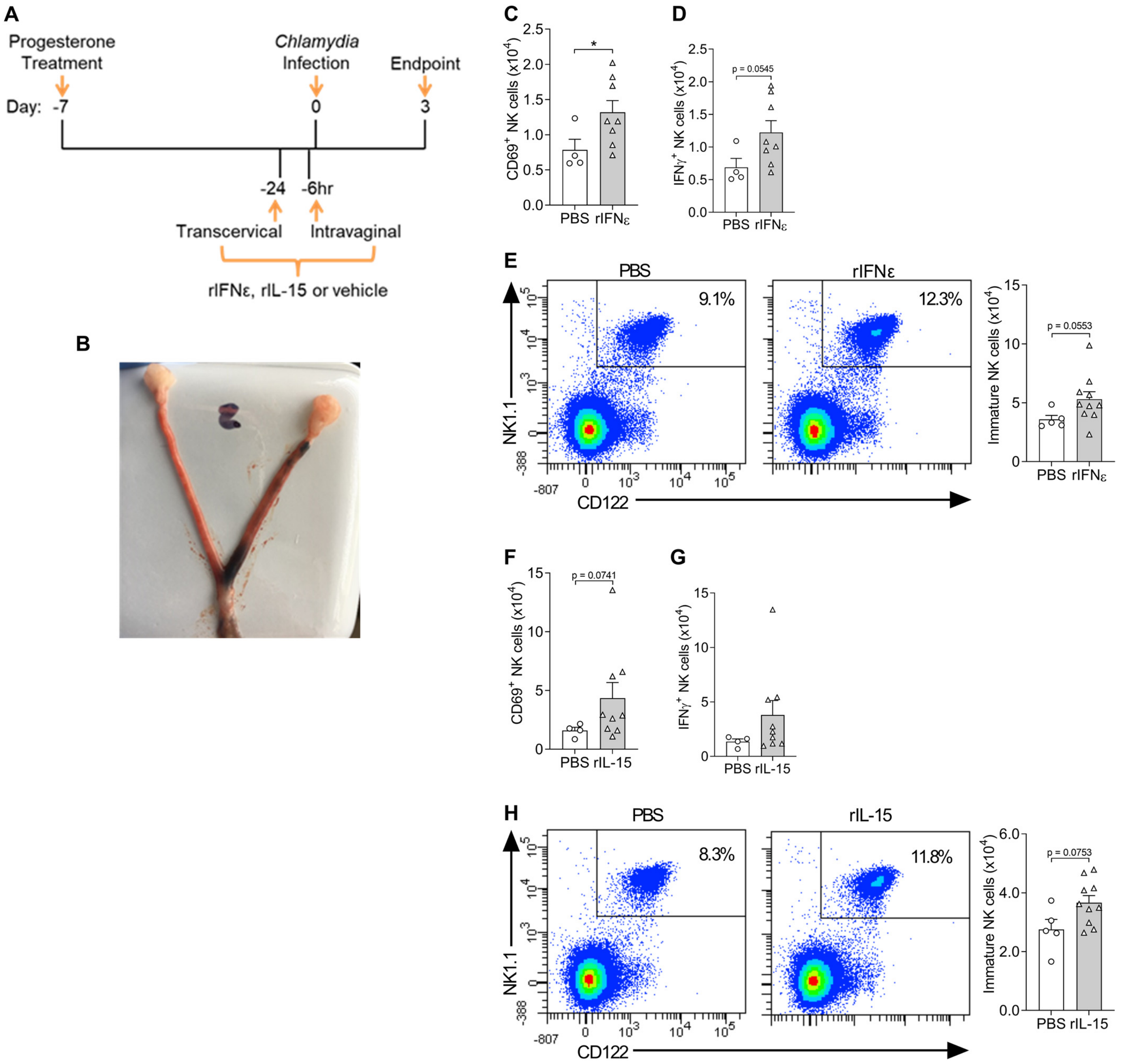

Figure EV4. In vivo administration and effect of recombinant (r)interferon (IFN)ε or rIL-15 on the numbers of activated and IFNγ-producing NK cells in the uterus and immature NK cells in bone marrow during *Chlamydia* infection.

(**A**) Seven days prior to infection mice were administered progesterone subcutaneously. Twenty-four hours prior to infection rIFNε, rIL-15 or phosphate-buffered saline (PBS))/PBS with 0.1% bovine serum albumin (BSA) vehicle was delivered into the uterine lumen using a small animal endoscope. Six hours prior to infection rIFNε, rIL-15 or vehicle was delivered into the vagina using a pipette. On day 0 mice were infected intravaginally with *C. muridarum*. At 3 days post infection, mice were culled and immune responses assessed. (**B**) Staining throughout uterus of mouse administered 100 μL Evan's blue dye transcervically demonstrating the distribution of substances delivered using this technique. (**C, D**) Quantification of (**C**) CD69$^+$ and (**D**) IFNγ$^+$ conventional NK cells (FSC$^{low\text{-}int}$ SSC^{low} CD45$^+$ CD3$^-$ NK1.1$^+$) in uterine horns from wild-type (WT) C57BL/6 mice prophylactically administered rIFNε or phosphate-buffered saline (PBS) vehicle control transcervically on day 3 of *Chlamydia muridarum* infection. (**E**) Flow cytometry of bone marrow from femurs as in A, showing immature conventional NK cells (FSC$^{low\text{-}int}$ SSC^{low} CD45$^+$ lin$^-$ CD11b$^-$ CD122$^+$ NK1.1$^+$) and quantification. (**F, G**) Quantification of (**F**) CD69$^+$ and (**G**) IFNγ$^+$ conventional NK cells (FSC$^{low\text{-}int}$ SSC^{low} CD45$^+$ CD3$^-$ NK1.1$^+$) in uterine horns from wild-type (WT) C57BL/6 mice prophylactically administered rIL-15 or 0.1% bovine serum albumin (BSA) in phosphate-buffered saline (PBS) vehicle control transcervically on day 3 of *Chlamydia muridarum* infection. (**H**) Flow cytometry of bone marrow from femurs as in (**D**), showing immature conventional NK cells (FSC$^{low\text{-}int}$ SSC^{low} CD45$^+$ lin$^-$ CD11b$^-$ CD122$^+$ NK1.1$^+$) and quantification. Data information: The % displayed on the flow cytometry plots are the % of the parent population the cells within the gates comprise. All data presented as mean ± SEM, with individual values. *$p < 0.05$ ((**A, B, D, E**): one-tailed Mann–Whitney test; (**C, F**): two-tailed Mann–Whitney test). (**C–E**): $n \geq 4$ (data from one experiment), (**F–H**): $n \geq 4$ (data from one experiment; all biological replicates).

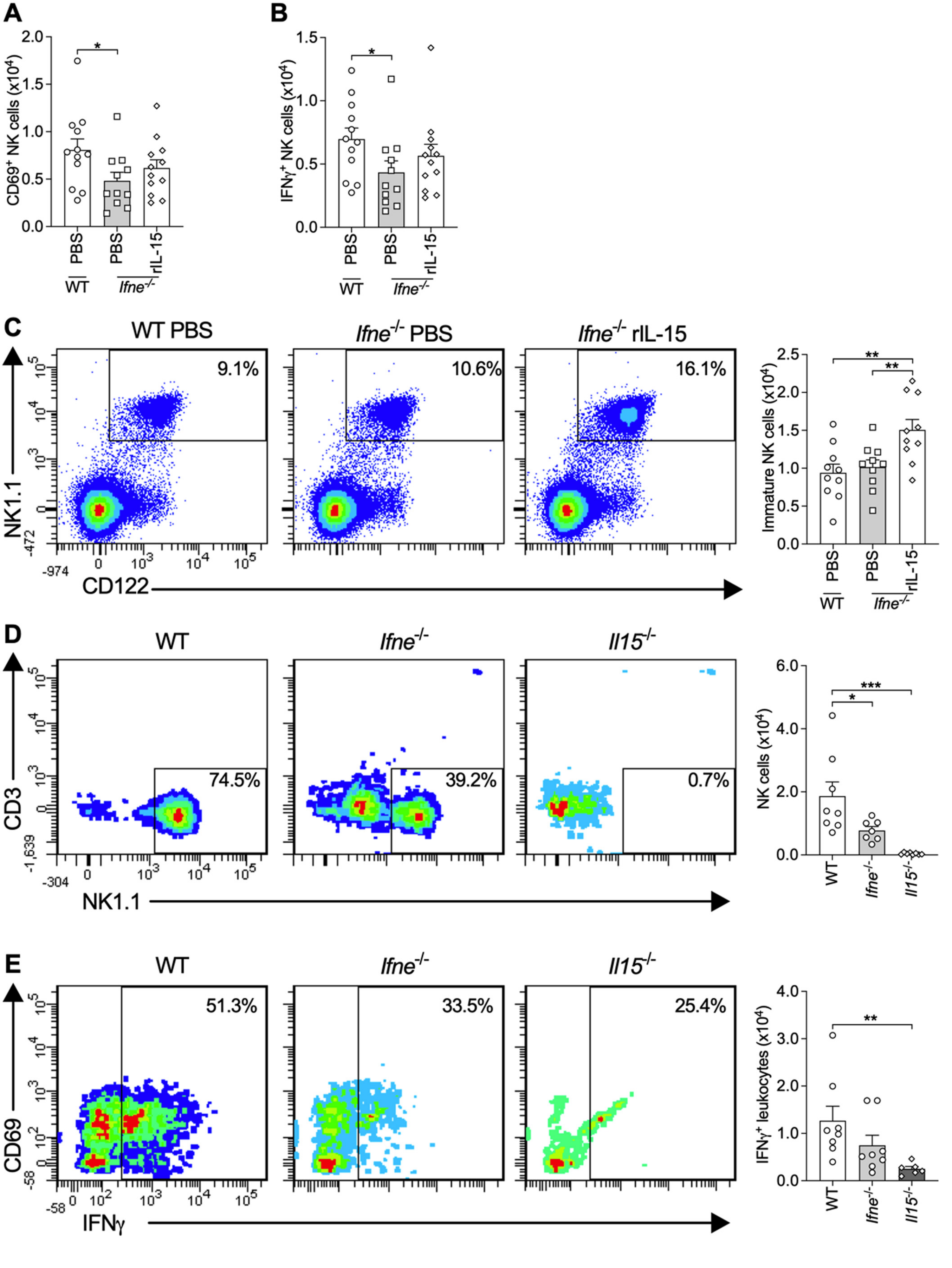
A
CD69+ NK cells (x10^4)
PBS
PBS
rIL-15
WT
Ifne-/-
B
IFNγ+ NK cells (x10^4)
C
WT PBS
Ifne-/- PBS
Ifne-/- rIL-15
9.1%
10.6%
16.1%
NK1.1
CD122
Immature NK cells (x10^4)
D
WT
Ifne-/-
Il15-/-
74.5%
39.2%
0.7%
CD3
NK1.1
NK cells (x10^4)
E
51.3%
33.5%
25.4%
CD69
IFNγ
IFNγ+ leukocytes (x10^4)

 © The Author(s)

Figure EV5. Recombinant (r)IL-15 has no effect on the numbers of activated and interferon (IFN)γ-producing NK cells in the uterus and increases immature NK cells in the bone marrow in *Ifne*-deficient ($^{-/-}$) mice and *Il15*$^{-/-}$ mice have no NK cells and reduced IFNγ^{+} leukocytes in the uterus during *Chlamydia* infection.

(**A**, **B**) Quantification of (**A**) CD69^{+} and (**B**) IFNγ^{+} conventional NK cells (FSC$^{low\text{-}int}$ SSC^{low} CD45^{+} CD3^{-} NK1.1^{+}) in uterine horns from *Ifne*$^{-/-}$ and wild-type (WT) C57BL/6 mice prophylactically administered rIL-15 (*Ifne*$^{-/-}$) or 0.1% bovine serum albumin (BSA) in phosphate-buffered saline (PBS) vehicle control (*Ifne*$^{-/-}$ and WT) transcervically on day 3 of *Chlamydia muridarum* infection. (**C**) Flow cytometry of bone marrow from femurs as in (**A**) showing immature conventional NK cells (FSC$^{low\text{-}int}$ SSC^{low} CD45^{+} lin^{-} CD11b^{-} CD122^{+} NK1.1^{+}) and quantification. (**D**) Flow cytometry of uterine horn cells from *Il15*$^{-/-}$, *Ifne*$^{-/-}$ and wild-type (WT) C57BL/6 mice on day 3 of *Chlamydia muridarum* infection showing conventional NK cells (FSC$^{low\text{-}int}$ SSC^{low} CD45^{+} CD3^{-} NK1.1^{+}) and quantification. (**E**) Flow cytometry of uterine horn cells, showing IFNγ^{+} leukocytes (CD45^{+} cells) and quantification. Data information: The % displayed on the flow cytometry plots are the % of the parent population the cells within the gates comprise. All data presented as mean ± SEM, with individual values. $*p < 0.05$, $**p < 0.01$, $***p < 0.001$ (one-way ANOVA). (**A**, **B**): $n \geq 11$, (**C**): $n = 10$ (data from one experiment), (**D**, **E**): $n = 8$ (data from one experiment; all biological replicates).

© The Author(s)
